# Design of a Verification Device of Motor Axle Wheel Load Scales Based on Pump-Controlled Hydraulic Cylinder

**DOI:** 10.3390/s25237180

**Published:** 2025-11-25

**Authors:** Long Hao, Zhipeng Xu, Bin Zhou, Gaoming Zhang

**Affiliations:** 1 Key Laboratory of In-Situ Measurement of Ministry of Education, China Jiliang University, Hangzhou 310018, China; 2 The State Key Laboratory of Fluid Power and Mechatronic Systems, Zhejiang University, Hangzhou 310027, China

**Keywords:** pump-controlled hydraulic, axle wheel load scales, verification device

## Abstract

Vehicle axle load scales are one of the most important devices for vehicle safety testing. To ensure the stability and reliability of test results, regular calibration of axle load scales is necessary. Traditional calibration methods are inefficient and error-prone. In this work, an automatic calibration device for portable axle load scales was presented, which uses a pump-controlled hydraulic cylinder as a loading unit. The loading unit was controlled by a high-precision force sensor and a PLC. A hydraulic unit based on a servo motor and a gear pump was designed, and control software including automatic control, data acquisition, and report generation was developed. The experimental test was carried out. The results showed that the developed portable automatic calibration device could realize the automatic calibration of a 0~150 kN load range, and the accuracy level was up to ±0.3%. Finally, it was verified that the device had the advantages of compactness and lightweight and simple operation.

## 1. Introduction

At present, motor vehicles have become one of the most important means of transportation for people to travel and transport goods. Trucks with overloaded axles represent a significant threat to traffic safety and may cause serious damage to the road surface [1]. Overloaded axles not only lead to increased erosion on the road surface, but also lead to an increased braking distance and more serious accidents due to higher impact energy. The overloading of axles can result in the overheating and failure of tires and brakes. If the permissible axle weight of a steering axle is exceeded, the steering is more cumbersome, which makes it possible to lose control of the vehicle [2]. Ensuring minimum axle load is essential for vehicle safety [3]. To ensure the safety of vehicles during travel and transportation, vehicle inspection stations and relevant overloading supervision units need to use special axle/wheel load scales for vehicle testing to detect the load of motor vehicle axles/wheels.

The special axle/wheel load scales for motor vehicle testing are instruments that weigh the axle load (or wheel load) of motor vehicles to determine the distribution of the axle load (or wheel load) of the motor vehicles. They can be divided into axle load scales and wheel load scales, and their verification has been conducted according to [4]. At present, the verification methods of vehicle test axle (wheel) load scales in China include a calibration weight verification method and a standard dynamometer verification method. The calibration weight verification method involves placing the standard calibration weight on the bearing platform of the equipment under testing and comparing the displayed value of the equipment under testing with the standard value of the calibration weight. The advantages of the calibration weight verification method are simple operation and reliable verification results, but the disadvantages lie in the large volume and heavy weight of the calibration weights required for verification and the cooperation of several people required to complete the verification. The standard dynamometer verification method uses a standard dynamometer instead of a calibration weight as the standard load reference. The traditional verification method requires manual pressure to load via the jack, a process that increases the intensity of the work and results in low verification efficiency. Meanwhile, manual counting in this method is susceptible to subjective factors, leading to recording errors. In the research, the verification method used for the verification device is the standard dynamometer verification method.

Weigh-in-motion (WIM) is a system used in Western countries to measure the gross weight of vehicles (gross vehicle weight—GVW). Currently, the WIM system is mainly applied to the weight inspection of vehicles passing over bridges and roads. This system directly installs force sensors on bridges and roads, and its detection principle is to measure the dynamic load of vehicles through the force sensors, thereby estimating the static load of each axle of the vehicle and the total weight of the vehicle [5,6]. WIM technology can provide various types of information about the traffic, including, but not limited to, vehicle class and speed, vehicle count, gross vehicle weight (GVW), wheel and axle weights, axle spacing, and the date and time of the event [7,8]. The precision of weigh-in-motion systems depends on the weighing error compared to standard values established through static measurements conducted during the station’s regular calibration. The advancement of WIM systems is progressing towards automated solutions, contingent on regulatory changes and improved system accuracy [9,10]. Ref. [11] mentions a calibration method for an automatic weighing instrument, which calibrates the WIM instrument by controlling the total vehicle weight and operating other detection equipment. Although it provides a calibration method, the operation is relatively complex. Ref. [12] developed a literature tree model, using on-site WIM information and sensor-related factors to estimate the expected range of WIM measurement errors, ensuring the accuracy of WIM data. Ref. [13] modeled the function of individual axle errors, and the test results showed that the calibration error was within the acceptable range and significantly reduced the calibration time. Ref. [14] proposed a metrological evaluation method for calibration reference values, maintaining the required precision while simplifying the calibration procedure. In summary, Western countries use the dynamic vehicle weighing system for vehicle axle weight detection, and they conduct research on the calibration methods of the WIM system to improve accuracy and shorten calibration time.

In China, since there are no dynamic weighing systems installed on bridges or roads, the axle–wheel load scale is used to measure vehicle weight. The verification of the axle–wheel load scale requires the use of an axle–wheel verification device. However, the traditional method of axle–wheel verification is labor-intensive and is prone to errors due to human factors, thereby affecting the efficiency and accuracy of the verification. With the development of automation technology, the traditional method of axle–wheel verification has also undergone technological changes. The hydraulic unit [15] has a strong load capacity and stable force output characteristics, making it suitable for use as the loading method in the axle–wheel verification device. A pump-controlled hydraulic unit is introduced into the axle load scales verification device. By replacing the jack in the traditional calibration device with the pump-controlled hydraulic unit, the intensity of the work will be greatly reduced. At the same time, control software has been designed to complete the automatic operation of the verification process and improve the degree of automation of the verification device. The verification data is synchronously recorded and saved, avoiding data errors caused by human interference. In this work, a set of automatic verification devices for axle/wheel load scales was designed with a pump-controlled hydraulic unit as the core, which can realize the automatic verification of axle/wheel load scales and data information management, improving verification efficiency.

## 2. Verification Device

According to the relevant testing standards, the axle load scale verification equipment can realize the automatic verification of axle load scales with a range of 0~150 kN; its accuracy class is ±0.3% of reading. In terms of portability, its total weight does not exceed 20 kg; additionally, the controller has dimensions of 900 mm (length) × 500 mm (width) × 200 mm (height).

**Figure 1:**
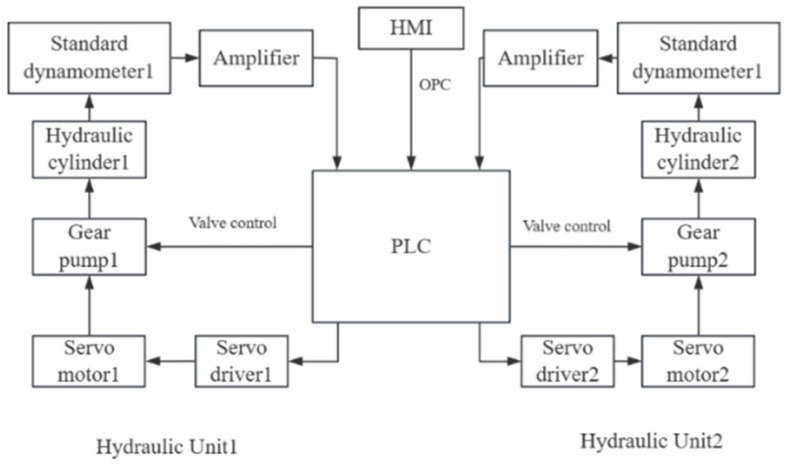
Verification device block diagram.

According to the relevant verification regulations, the axle load scale requires multiple force loading devices to take simultaneous measurements during the verification process [1]. Therefore, the verification device needs at least two sets of hydraulic units. The verification device of axle/wheel load scales is composed of a control unit and a hydraulic mechanism, in which the control unit consists of an industrial computer, a PLC, an amplifier, and two servo drivers. The hydraulic mechanism consists of a servo motor, a gear pump, two reversing valves, a cylinder, a tank, a relief valve, an accumulator, and a pressure sensor. The verification device block diagram is shown in Figure 1. The main function of the verification device is as follows. The virtual instrument is connected to the PLC through OPC; the PLC is connected to the servo motor, which then drives the hydraulic unit; pressure is then applied to the equipment under test via the anti-gravity frame; and finally, the pressure sensor measures the pressure data, and this process realizes the system’s pressure measurement and pressure relief. Firstly, the standard value of the verifying equipment (standard device) is compared with the indicated value of the inspected equipment (equipment under test); the relevant data is then recorded. Subsequently, the error is calculated and, finally, a verification report is generated. The verification data upload function is realized through the Laboratory Information Management System (LIMS).

### 2.1. Working Principle

**Figure 2:**
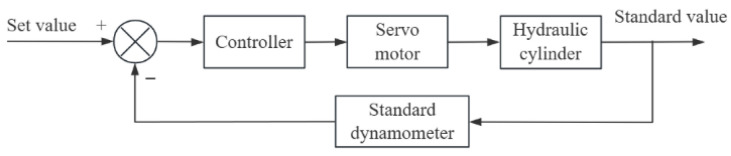
Standard value closed-loop feedback schematic diagram.

To ensure that the output value of the standard dynamometer can remain stable at the set target value, feedback on the output value is required to guarantee the accuracy and reliability of the output value of the standard dynamometer. The feedback loop schematic diagram of the verification device for the standard value is shown in Figure 2. The target value is set and transmitted to the servo motor through the control software to drive the hydraulic unit to operate, thereby outputting the standard value. The difference between the output standard value and the set value is calculated, and based on this difference, the control software dynamically adjusts the signal transmitted to the servo motor to ensure that the loading force output by the hydraulic cylinder reaches the target value. First of all, according to the verification standard, the test is selected on the control software of the industrial computer to determine the corresponding set value. The range includes 10%FS, 20%FS, 50%FS, and 100%FS; this is to achieve the required pressure target value. Then, the hydraulic unit is started for pressurization; the loading force value is detected by the pressure sensor, fed back to the control software, and used to provide the standard force value required for the verification process.

The pressure signal from the pressure sensor is amplified by the transmitter and converted into a digital signal, which is then fed back to the industrial computer. The standard force value is subsequently displayed on the control software. The load value of the tested equipment is synchronously displayed on the control software, and the calibrator records the measurement data via the control software. After the measurement data is collected, a report is generated, followed by pressure relief.

### 2.2. Hydraulic Unit

**Figure 3:**
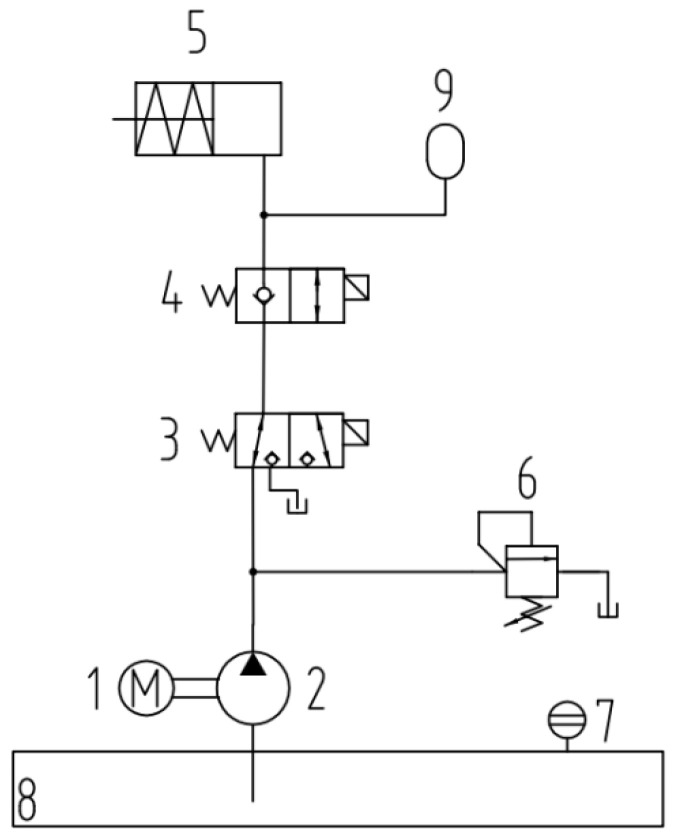
Schematic diagram of hydraulic unit: (1) motor, (2) gear pump, (3) two-position three-way reversing valve, (4) two-position two-way reversing valve, (5) cylinder, (6) direct-acting relief valve, (7) vision mirror, (8) tank, (9) accumulator.

The core part of the verification device is the hydraulic unit, which provides pressure. The schematic diagram of the hydraulic unit is shown in Figure 3. The hydraulic unit is powered by a gear pump driven by a servo motor, and the hydraulic oil enters the hydraulic cylinder through the reversing valve to drive the actuator to move. The accumulator functions to ensure the stability of the system’s hydraulic oil pressure, while the direct-acting relief valve acts as a safety valve in the device, discharging excess hydraulic oil back to the oil tank. Once the test data is collected, the motor stops running, and the reversing valve is actuated to relieve pressure, allowing the hydraulic oil to be discharged back to the oil tank. Table 1 shows the specifications of the key components of the hydraulic unit.

The hydraulic system is divided into no-load operation and loading operation during operation. The motor speed is the same as the gear pump speed under no-load conditions.

During loading, the loading force is formed by the pressure from the anti-gravity frame, and the loading force F is calculated by Formula (1).

(1) $$F=P\cdot \pi {\left(\frac{\phi }{2}\right)}^{2}$$

In the formula, F represents the loading force, with the unit being Newtons (N); P represents pressure, with the unit being Pascal (Pa); and ϕ represents the cylinder diameter, with the unit being meters (m).

The maximum force required for axle load verification is 150 kN. With the cylinder diameter basically determined as 100 mm, the calculated pressure is at least 19.1 MPa; the design pressure of standard parts and custom parts must exceed this pressure. Referring to the typical pressure of a hydraulic system, the maximum working pressure of the pump and other hydraulic components is determined to be 25 MPa. The cylinder radius is 50 mm, which is substituted into Formula (1) to calculate a loading force of 196,250 N. It can be seen from the calculation results that this loading force meets the 150,000 N requirement.

The loading speed was calculated by Formula (2).

(2) $$v=\frac{n\cdot V}{\pi ({\frac{\phi }{2})}^{2}}\;$$

In the formula, v represents the speed, with the unit being meter per second (m/s); n represents rotational speed, with the unit being revolutions per second (r/s); and V represents the pump delivery, with the unit being cubic meters per second (m^3^/s).

**Figure 4:**
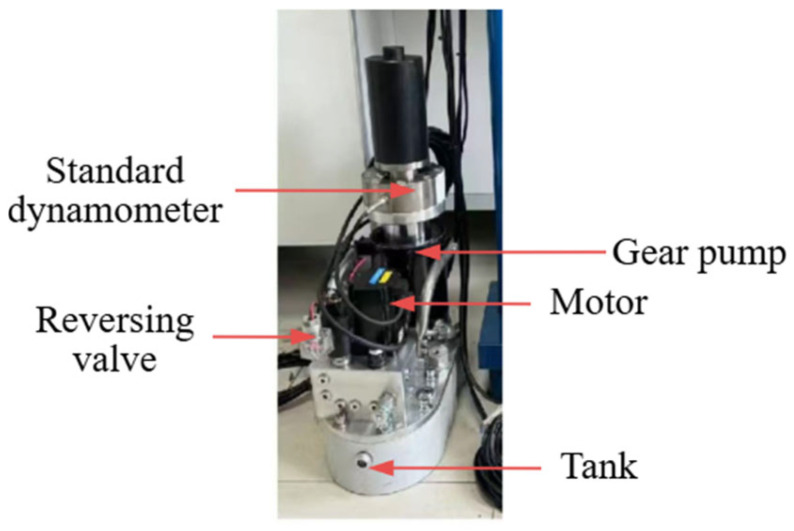
Physical diagram of hydraulic unit.

**Figure 5:**
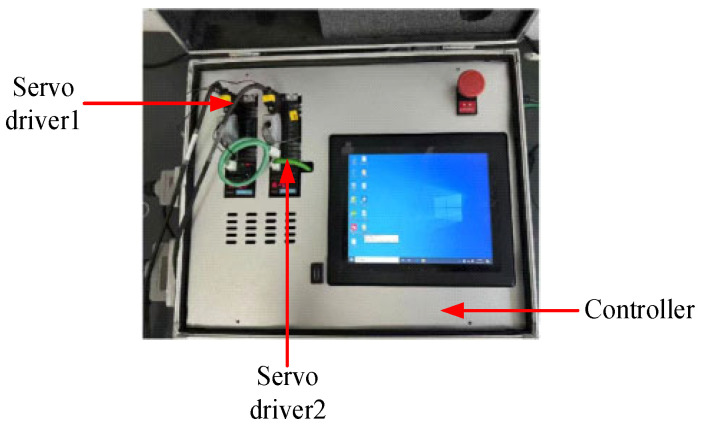
Physical diagram of control box.

The no-load speed and the loading speed can be manually set on the upper computer according to the actual situation. The physical diagram of the hydraulic unit is shown in Figure 4. The physical diagram of the control box is shown in Figure 5.

#### Modeling of Pump-Controlled Hydraulic Unit

**Figure 6:**
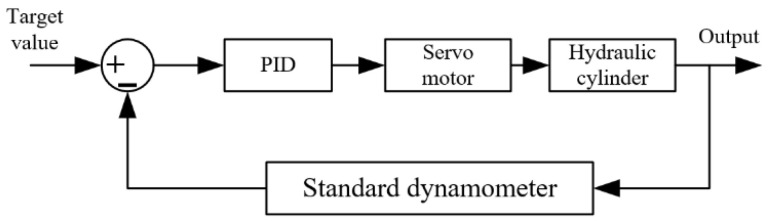
Schematic diagram of PID controller.

To ensure that the output of the hydraulic unit reaches the accurate target value, the hydraulic unit is regulated through a PID controller. The schematic diagram of the PID controller is shown in Figure 6.

The output torque of the servo motor drives the pump to rotate [16], and the motion equation is as follows:

(3) $$J_{m}\frac{{d\omega }_{m}}{dt}=T_{m}-T_{Lm}-B_{m}\omega_{m}\;$$

In the formula, $J_{m}$ is motor rotational inertia (kg·m^2^); $\omega_{m}$ is motor angular velocity (rad/s); $T_{m}$ is motor electromagnetic torque (N·m); $T_{Lm}$ is the load torque of the pump (N·m); and $B_{m}$ is the motor viscous friction coefficient (N·m·s/rad).

(4) $$T_{Lm}=\frac{D_{p}p_{L}}{2\pi }\;$$

In the formula, $D_{p}$ is pump displacement (m^3^/rad); $p_{L}=p_{1}-p_{2}$ is load pressure (Pa); and $p_{1}$, $p_{2}$ are the pressure of the two chambers of the hydraulic cylinder.

The electromagnetic torque of the motor is directly proportional to the control voltage u (servo amplifier gain $K_{a}$).

(5) $${T_{m}=K}_{a}u$$

Formulas (3)–(5) are combined after Laplace transformation:

(6) $$\Omega_{m}\left(s\right)=\frac{K_{a}}{J_{m}s+B_{m}}U\left(s\right)-\frac{D_{p}}{2\pi \left(J_{m}s+B_{m}\right)}P_{L}\left(s\right)\;$$

The output flow equation of the pump must now be formulated [17]. The output flow of the pump is directly proportional to the angular velocity of the motor. Taking into account the leakage loss, the pump’s output flow $Q_{p}$ is represented as follows:

(7) $$Q_{p}=D_{p}\omega_{m}$$

After Laplace transformation:

(8) $$Q_{p}\left(s\right)=D_{p}\Omega_{m}\left(s\right)$$

Next is the hydraulic cylinder continuity equation [18]. The two chambers of the hydraulic cylinder comply with the continuity principle. Taking into account the compressibility of the oil, the flow equation is as follows:

(9) $$Q_{p}=A\frac{dx}{dt}+\frac{V_{t}}{4K_{e}}\frac{dp_{L}}{dt}$$

In the formula, A is effective area of the hydraulic cylinder piston (m^2^); x is piston displacement (m); $V_{t}$ is the total volume of the two chambers of the hydraulic cylinder (m^3^); and $K_{e}$ is volumetric elastic modulus of oil liquid (Pa).

After Laplace transformation:

(10) $$Q_{p}\left(s\right)=AsX\left(s\right)+\frac{V_{t}}{4K_{e}}sP_{L}\left(s\right)$$

The output force of the hydraulic cylinder overcomes the inertial load, viscous damping load, and external load force, and the force balance equation is as follows [19]:

(11) $$Ap_{L}=M_{t}\frac{d^{2}x}{dt^{2}}+B_{p}\frac{dx}{dt}+Kx+F_{L}$$

In the formula, $M_{t}$ is the mass of the piston and the load together (kg); $B_{p}$ is the load viscous damping coefficient (N·s/m); K is load elastic stiffness (N/m); and $F_{L}$ is the external load force (N).

After Laplace transformation:

(12) $$AP_{L}\left(s\right)=\left(M_{t}s^{2}+B_{p}s+K\right)X\left(s\right)+F_{L}\left(s\right)$$

After combining Formulas (6), (8), (10), and (12), ignoring the load elasticity (K=0) and viscous damping, $B_{p}$ approaches zero. Furthermore, disregarding the higher-order small terms (since the motor inertia is relatively small), and with the inertia load being dominant, it is simplified to a second-order system:

(13) $$G\left(s\right)=\frac{X(s)}{U(s)}=\frac{K\omega_{n}^{2}}{s^{2}+2\zeta \omega_{n}s+\omega_{n}^{2}}$$

(14) $$K=\frac{2\pi K_{a}A}{D_{p}}\;$$

(15) $$\omega_{n}=\sqrt{\frac{\pi A^{2}V_{t}}{2D_{p}^{2}K_{e}M_{t}}}$$

(16) $$\zeta =\frac{4\pi B_{m}K_{e}M_{t}+D_{p}^{2}V_{t}}{8\pi K_{e}M_{t}\sqrt{\frac{\pi A^{2}V_{t}}{2D_{p}^{2}K_{e}M_{t}}}}\;$$

The parameters are shown in Table 2. Substitute the parameters from Table 2 into Formula (16) and obtain Formula (17).

(17) $$G\left(s\right)=\frac{749.16}{1000s^{2}+0.381s+0.004}$$

**Figure 7:**
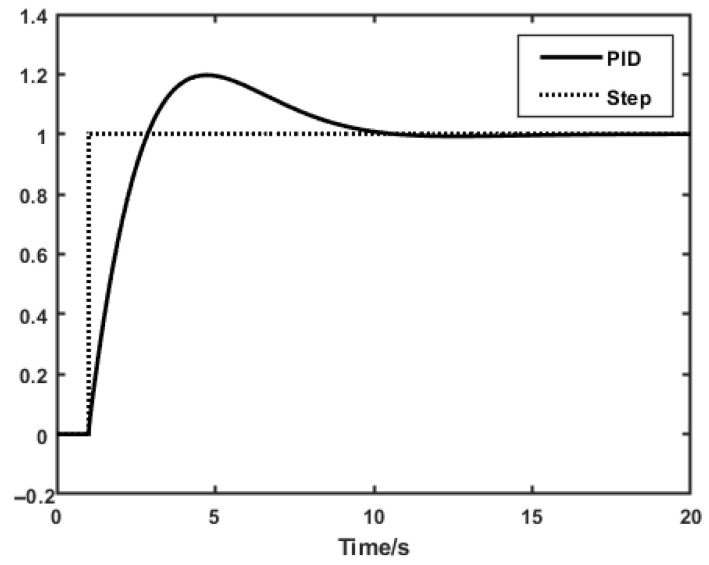
The simulation graph.

Through simulation analysis using MATLAB R2018b/ Simulink, after parameter adjustment, P = 30, I = 12, and D = 35. After inputting a step signal, the PID curve approached the target value at 10 s. The simulation graph is shown in Figure 7.

### 2.3. Control Software

Software requirements include a display control module, a data acquisition module, a report generation module, and a LIMS upload module. The system realizes communication between the host computer and the PLC through OPC configuration, and the PLC controls the servo motor, the reversing valve, and the no-load/loading speed. The verification device communicates with the tested device via RS232 and reads and records the display value in real time. Reports are automatically generated based on all parameter measurements of the equipment to be tested, and the report information is uploaded to the LIMS of the Metrology Institute. The LIMS can also obtain the basic information about the equipment to be tested.

**Figure 8:**
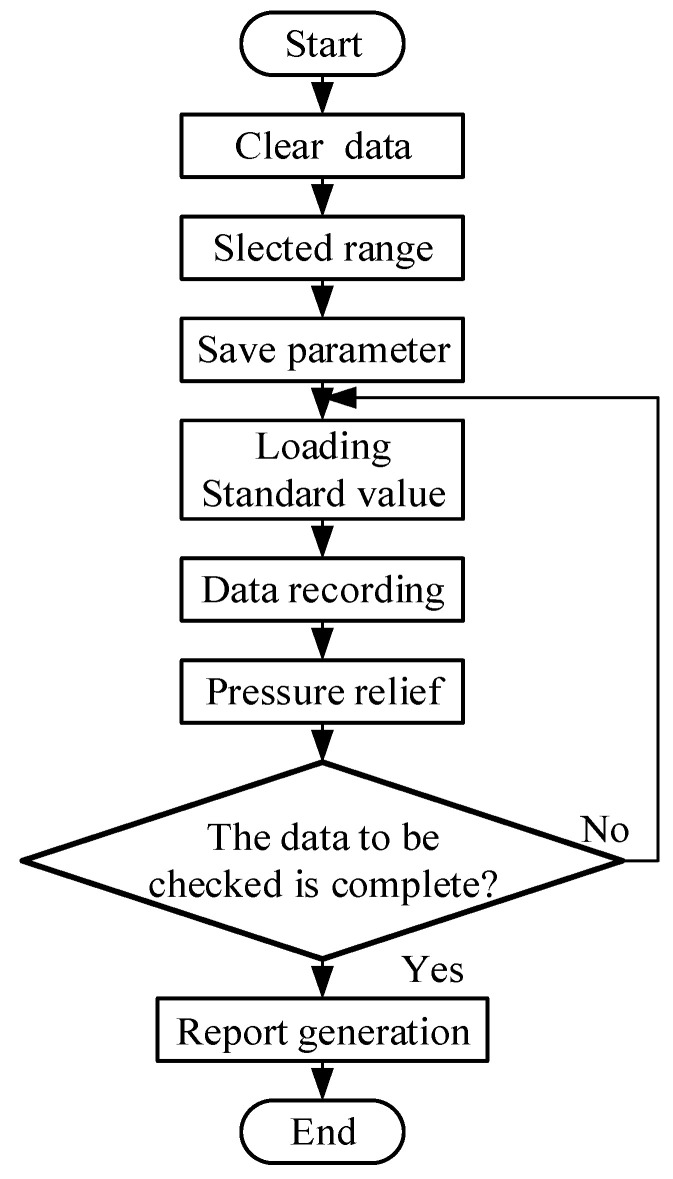
Software execution flow.

The software execution flow is shown in Figure 8. When the verification software is run, the existing data in the table is automatically cleared; after that, the measuring range of the tested equipment is selected, and the standard force value for the required measuring point is selected in accordance with the verification regulations. After the parameters are saved, the hydraulic system of the equipment starts to load; after loading to the standard force value, the displayed value of the tested equipment is recorded. Then, after the data is recorded, pressure relief is performed before restarting, and verification of the next measuring point begins. Finally, when all measuring points have been verified, a report is generated, uploaded to the LIMS, and the verification software is exited.

## 3. Experimental Test and Uncertainty Evaluation

### 3.1. Experimental Test

**Figure 9:**
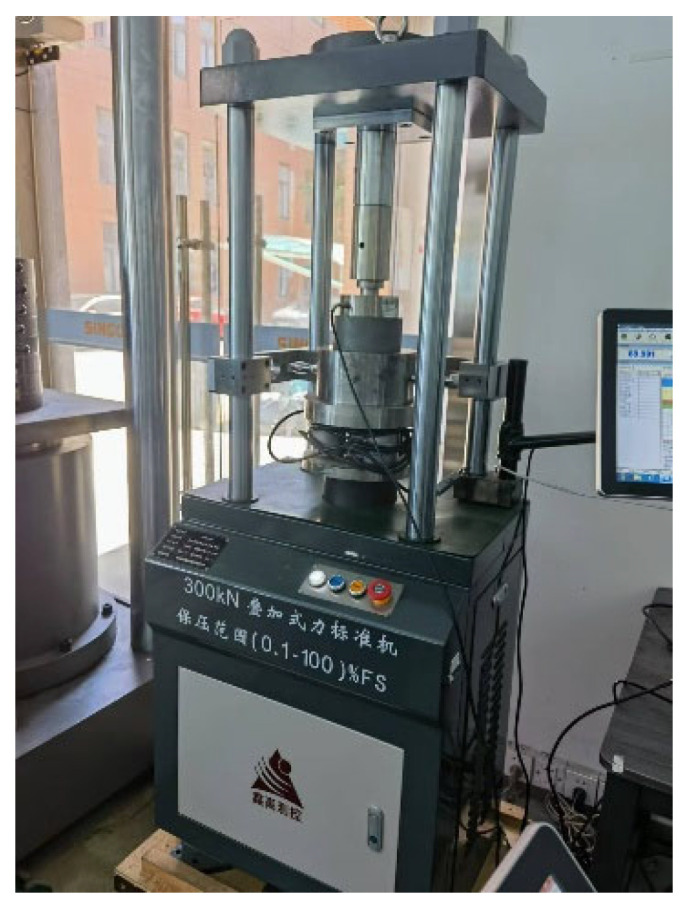
Physical diagram of standard dynamometer and loading verification device.

In order to ensure the reliability and accuracy of the verification device’s data when verifying axle load scales, it is necessary to verify the two sets of standard dynamometers in the device after the design is completed. The physical diagram of the standard dynamometer and the loading verification is shown in Figure 9. In the verification process of the standard dynamometer, a certain error is found between the measured data and the standard value; to correct this error, the dynamometer needs to be calibrated. The linear interpolation method is used to calibrate the standard dynamometer. After calibration, the standard dynamometer was verified again, and the verification results met the requirements. Table 3 shows the calibration data of the standard dynamometer.

Through the one-dimensional stability analysis outlined in [20], it is known that the accumulator has an inhibitory effect on pressure fluctuations, and this conclusion rules out the one-sided view that “pressure drop is caused solely by pump leakage”. In the study of [21], researchers conducted experiments on parameters such as the linear velocity of the hydraulic cylinder and the connection effect between the accumulator and the system, further verifying the key role of the accumulator in maintaining stability in hydraulic systems. In addition, by studying the pressure dynamic characteristics of the piezoelectric pump system with a one-way valve in [22], it was found that the combination of a closed flow path and an accumulator can effectively reduce pressure fluctuations caused by the rapid closure of the valve; specifically, the pressure slightly decreases within a short period (<300 s) and then tends to stabilize. Based on the above-mentioned studies, it can be seen that such short-term pressure drops occurring in pump-controlled hydraulic units are a normal phenomenon, and this characteristic also indirectly proves that this hydraulic unit has good stability.

**Figure 10:**
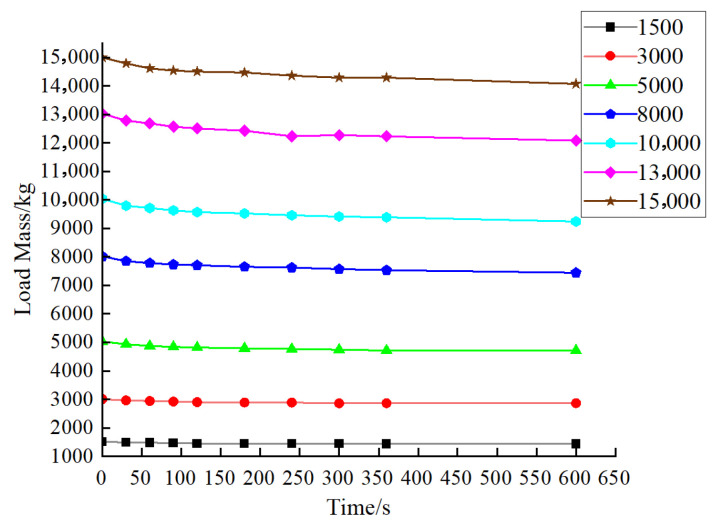
Stability experimental data curve of Hydraulic Unit 1.

**Figure 11:**
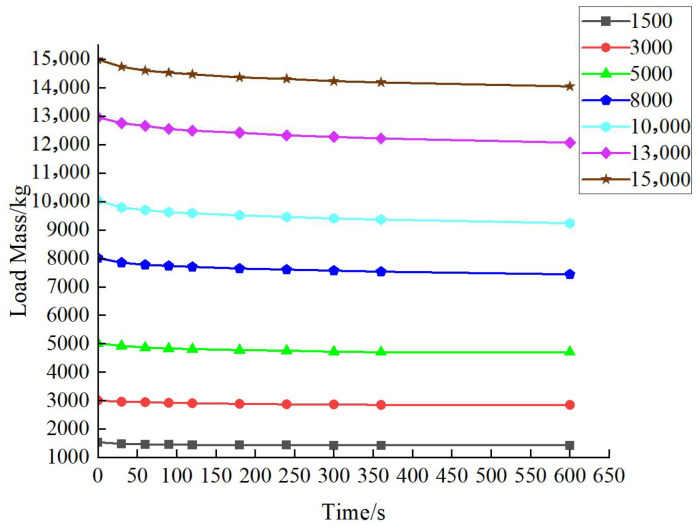
Stability experimental data curve of Hydraulic Unit 2.

After the calibration of the standard force value. It is necessary to conduct experimental tests on the stability of Hydraulic Unit 1 and Hydraulic Unit 2 of the verification system. After loading to the measuring point via the loading device, we observe the changes in the tested equipment’s measuring point data and record the corresponding data. Table 4 and Table 5 show the experimental data on the stability of Hydraulic Unit 1 and Hydraulic Unit 2, and Figure 10 and Figure 11 show the curves of their experimental stability data.

The experimental results show that the stability is maintained for a relatively long period within the range of 30 s to 90 s, which can meet the reading requirements for the verification. Moreover, the downward trend becomes more gradual over time. Overall, the hydraulic unit has excellent pressure retention performance.

To ensure the reliability of the verification device’s data, a repeatability test needs to be conducted on the verification device. For this, the load force was set to 20% FS, meaning that the load mass is 3000.0 kg and was then measured three times [23]. The test data are shown in Table 6. By calculating according to Formula (18), the repeatability can be obtained.

(18) $$R=\frac{X_{max}-X_{min}}{X}\times 100\%$$

In the formula, R represents the repeatability, $X_{max}$ represents the maximum value, $X_{min}$ represents the minimum value, and X represents the standard value.

According to the procedure, the repeatability of the axle load scale should not exceed half of the absolute value of the maximum allowable error at the inspection point. The absolute value of the maximum allowable error at 20% FS is 20 kg, and the repeatability should not exceed 10 kg, which is 0.33% [1]. The experimental data shows that the repeatability of the hydraulic unit is 1 kg, which is one-tenth of the axle load scale, so the repeatability of the hydraulic unit is good and can meet the measurement requirements of the axle load instrument.

### 3.2. Uncertainty Evaluation

The uncertainty introduced by the standard dynamometer calibration device is 0.05 grade, with the accuracy level of the calibration device being of uniform distribution. At the measurement point of 150 kN, the standard uncertainty *u*_1_ is introduced by the calibration device, as shown in Formula (19):

(19) $$u_{1}=\frac{150\;kN\times 0.05\%}{\sqrt{3}}=0.043\;kN$$

The uncertainty introduced by the standard force measuring instrument is 0.3 grade. The accuracy level of the standard force measuring instrument is 0.3 grade, the upper limit of the measurement range is 150 kN, and the measured error follows a uniform distribution. Then, the standard uncertainty *u*_2_ introduced by the standard force measuring instrument, as shown in Formula (20), is as follows:

(20) $$u_{2}=\frac{150\;kN\times 0.3\%}{\sqrt{3}}=0.260\;kN$$

The standard uncertainty introduced by the repeatability test of the force loaded by the hydraulic unit is a standard deviation of a single measurement; that is, the repeatability standard uncertainty *u*_3_ is 0.006 kN.

The synthetic standard uncertainty is *u*_c_.

(21) $$u_{c}=\sqrt{u_{1}2+u_{2}2+u_{3}2}=0.264\;kN$$

The expanded uncertainty is *U*, and *k* = 2.

(22) $$U=ku_{c}=0.528\;kN$$

## 4. Conclusions

The traditional method relies entirely on the manual operation of hydraulic jacks, which is labor-intensive, inefficient, and suffers from unstable loading processes. A key innovation of this study is the design of an automated closed-loop control system based on force sensor feedback.

This system automatically controls and stabilizes the loading force at the target value, fundamentally eliminating human error and significantly improving verification efficiency and reliability.

The verification regulation requires two loading devices to operate synchronously. Traditional manual methods can hardly ensure highly consistent and stable loads at both loading points, which is a critical bottleneck restricting verification accuracy and reliability.

This study achieves the centralized, synchronous, and precise control of two independent hydraulic units (Hydraulic Units 1 and 2) through integrated PLC control.

Experimental data indicate that both units exhibit excellent and consistent stability and repeatability (repeatability of 0.033%), successfully overcoming this key engineering challenge.

This study successfully integrates an electro-hydraulic servo control system, typically used in large industrial equipment, into a portable device with a total weight not exceeding 20 kg, achieving a reading accuracy of ±0.3%. This balance achieved among portability, accuracy, and automation level is in itself a significant engineering application innovation.

In summary, the innovativeness of this device is primarily demonstrated at the engineering methodology level; through automated and integrated design, it specifically addresses the inherent shortcomings of traditional methods in terms of efficiency, accuracy, and reliability, offering the industry a superior practical solution.

## Figures and Tables

**Table 1:** Key parameters of hydraulic unit.

Component Name	Type Specification
Servo Motor	400 W 3000 RPM
Gear Pump	0.5 cc/r
Reversing Valve3	20 L/min
Reversing Valve4	18.9 L/min
Overflow Valve	10 L/min
Accumulator	0.32 L
Hydraulic Cylinder	ϕ100 mm

**Table 2:** Parameters.

Parameters	Data
$K_{a}$	3
A	7.854 × 10^−3^ m^2^
$D_{p}$	7.96 × 10^−7^ m^3^/rad
$V_{t}$	9.483 × 10^−4^ m^3^
$K_{e}$	1.5 × 10^1^ Pa
$M_{t}$	2.4 kg
$B_{m}$	3.8 × 10^−4^ N·m·s/rad

**Table 3:** Calibration value of standard dynamometer after calibration.

Standard Value/N	Standard Dynamometer Calibration Value	
	Value1/N	Value2/N
0	0	0
15,000	15,027	15,040
30,000	30,083	30,090
45,000	45,020	45,070
60,000	59,967	60,050
75,000	74,943	75,040
90,000	89,917	90,040
120,000	119,860	120,000
150,000	149,860	149,940

**Table 4:** Stability test data of Hydraulic Unit 1.

Time/s	Load Mass/kg						
	1500	3000	5000	8000	10,000	13,000	15,000
0	1519.0	3006.2	5023.0	8016.0	10,033.0	13,020.0	14,995.0
30	1486.1	2960.6	4935.4	7849.2	9793.0	12,779.5	14,785.3
60	1475.6	2939.5	4872.0	7779.3	9709.0	12,680.5	14,608.0
90	1470.5	2920.5	4838.5	7732.1	9630.5	12,563.4	14,532.0
120	1448.0	2900.3	4819.7	7705.4	9566.2	12,503.1	14,500.0
180	1448.4	2885.4	4790.3	7649.5	9520.5	12,420.8	14,464.0
240	1448.3	2885.4	4765.4	7621.7	9455.0	12,231.0	14,356.0
300	1448.2	2860.5	4740.8	7570.4	9411.5	12,269.0	14,285.0
360	1430.6	2860.8	4712.0	7532.4	9386.4	12,224.0	14,285.0
600	1430.6	2860.8	4712.0	7443.6	9238.0	12,080.0	14,069.0

**Table 5:** Stability test data of Hydraulic Unit 2.

Time/s	Load Mass/kg						
	1500	3000	5000	8000	10,000	13,000	15,000
0	1536.2	3008.0	5017.0	8017.0	10,035.0	12,965.0	14,995.0
30	1481.1	2962.2	4925.1	7851.0	9794.0	12,757.7	14,739.0
60	1462.7	2943.8	4869.8	7776.7	9702.7	12,664.9	14,609.0
90	1462.7	2925.3	4832.4	7739.8	9628.6	12,554.3	14,535.0
120	1444.2	2906.9	4813.9	7702.9	9591.0	12,498.4	14,479.0
180	1444.2	2888.5	4777.0	7647.0	9517.1	12,424.6	14,368.0
240	1443.7	2870.0	4758.6	7610.0	9461.8	12,331.8	14,313.0
300	1425.8	2865.5	4721.7	7573.2	9406.5	12,276.5	14,238.0
360	1425.8	2851.6	4703.2	7535.8	9369.0	12,220.6	14,192.0
600	1425.8	2851.6	4703.2	7443.6	9239.4	12,072.5	14,053.0

**Table 6:** Repetitive test data.

Hydraulic Unit	Data1/kg	Data2/kg	Data3/kg	Repeatability
1	3006.0	3005.0	3006.0	0.033%
2	3008.0	3008.0	3009.0	0.033%

## Data Availability

Data are not publicly available and can be obtained by contacting the corresponding author if necessary.
